# Impact of Drinking Water Supplemented with Complex Acidifiers on Production Performance, Egg Quality, Physiological and Biochemical Indicators, and Microbial Flora of BIAN Chickens

**DOI:** 10.3390/life15111700

**Published:** 2025-11-03

**Authors:** Bochi Zhang, Liying Du, Tao Yu, Kai Zhang, Rui Zhao, Chunlei Yang, Xianyi Song

**Affiliations:** College of Animal Science, Shanxi Agricultural University, Taiyuan 030032, China; bochi_zhang@sxau.edu.cn (B.Z.); duliying@sxau.edu.cn (L.D.); 17710619040@163.com (T.Y.); zhangkai2255@126.com (K.Z.); zhaorui@sxau.edu.cn (R.Z.); wkyhlycl@126.com (C.Y.)

**Keywords:** compound acidifiers, BIAN chicken, production performance, intestinal development, nutritional regulation

## Abstract

This study investigated the effects of dietary supplementation with composite acidifying agents containing 2-hydroxy-4-methylthiobutyric acid (≥30.0%), lactic acid (≥24.2%), and phosphoric acid (≥23.8%) on production performance, egg quality, serum biochemistry, intestinal health, and cecal microbiota in 300-day-old BIAN chickens. In a 42-day randomized trial, 900 laying hens were randomly allocated to three groups: the control group (basal diet with tap water), test group A (basal diet with 0.05% composite acidifier in drinking water), and test group B (basal diet with 0.20% composite acidifier in drinking water). The results demonstrated that test group B exhibited a significant 4.6% increase in average egg weight compared to the control (*p* = 0.029), while test group A showed enhanced Haugh unit values (*p* = 0.010) and eggshell strength (*p* = 0.010). Serum biochemical analysis revealed marked improvements in immune function, with test group B showing a 65.49% increase in globulin levels (*p* = 0.010) and 61.76% elevation in total antioxidant capacity (T-AOC) (*p* = 0.010). Intestinal digestive enzyme activities were significantly enhanced, particularly in test group A with a 61.73% increase in duodenal lipase activity (*p* = 0.010) and 37.43% elevation in jejunal amylase activity (*p* = 0.036). Morphological assessment demonstrated improved intestinal architecture in test group B, with a 26.02% reduction in crypt depth (*p* = 0.025) and a 44.53% increase in the villus-to-crypt ratio (*p* = 0.030). Microbiota analysis revealed dose-dependent modulation of cecal bacterial communities, with notable increases in beneficial genera including *Akkermansia* (from 1.8% to 7.2% in test group A) and Lachnospiraceae (from 4.7% to 9.7% in test group B) while maintaining core microbiota stability. Principal component analysis confirmed distinct microbial ecological niches created by acidifier supplementation. These findings demonstrate that composite acidifying agents effectively enhance egg production quality, immune status, digestive function, and gut health in BIAN chickens, supporting their potential as sustainable alternatives to antibiotic growth promoters in laying hen production systems.

## 1. Introduction

The use of acidifiers in poultry nutrition has become an essential strategy to improve production performance and animal health. Organic acids, such as malic, citric, formic, lactic, and butyric acids, have demonstrated positive effects on broiler performance, enhancing feed conversion ratios, nutrient digestibility, and growth metrics [1,2,3,4]. The mechanisms underlying these performance improvements are closely linked to the acidifiers’ profound effects on the intestinal microbiota composition, as demonstrated by sodium diformate supplementation modulating beneficial bacteria populations while reducing pathogenic species [5] and phosphoric and lactic acids significantly decreasing E. coli and Salmonella counts in broiler cecum [6]. Drinking water acidification has emerged as an effective delivery method, with studies reporting that water acidification improved bone metabolism through altered serum biochemical indicators including increased phosphorus concentrations and reduced inflammatory markers [7] while demonstrating significant gastrointestinal pH reductions and improved feed conversion efficiency [8]. The effects of acidifiers extend to egg quality parameters, with recent studies showing enhanced Haugh unit scores and shell thickness in acidifier-supplemented laying hens [9], supported by documented improved egg yolk composition with combined probiotic–acidifier treatments [10]. Furthermore, recent studies have shown that acidifiers also demonstrate excellent effects in regulating the intestinal microbial flora and controlling pathogens in laying hens [11,12,13,14]. Contemporary research has increasingly focused on innovative formulations, including microencapsulated organic acid systems that provide targeted intestinal delivery [15] and synergistic combinations with probiotics and botanicals that enhance overall efficacy while maintaining intestinal homeostasis [16,17].

Recent advances in antibiotic-free poultry production have intensified research on feed additives that enhance growth performance and gut health, with 2-hydroxy-4-methylsulfonylbutyric acid (HMTBa), lactic acid, and phosphoric acid emerging as promising alternatives [18]. Phosphoric acid presents a unique dual functionality as both an acidifier and a phosphorus source, with studies showing 93% relative bioavailability and optimal inclusion at 0.1–0.2 g/kg for improved feed conversion and intestinal enzyme activities [18]. Meta-analyses encompassing 93 studies confirmed lactic acid bacteria’s positive effects on growth performance and immune responses [19], with recent trials demonstrating effectiveness in mitigating high-stocking-density stress [20]. Lactic acid has shown consistent antimicrobial benefits, with encapsulated forms at 0.6% inclusion significantly reducing Salmonella counts and enhancing beneficial bacteria populations [21], while strain-specific Lactobacillus plantarum applications increased beneficial microbiota and decreased pathogenic Desulfovibrio [22]. Multiple investigations have established HMTBa’s superior performance at higher inclusion levels with reduced negative impact on feed intake [23,24]. HMTBa has demonstrated 60–80% relative bioavailability compared to DL-methionine while offering unique metabolic advantages through concentration-dependent absorption mechanisms [25,26], with studies confirming its dual functionality in promoting beneficial butyrate-producing bacteria while reducing pathogens [27]. Despite these advances, significant research gaps persist, particularly regarding phosphoric acid applications in modern production systems and the synergistic effects of combined acidifier strategies under current high-density, fast-growth conditions.

The BIAN chicken, an indigenous breed in Shanxi Province, China, is notable for its moderate body size, distinctive feather pattern, and adaptability to the semi-intensive production system typical of traditional poultry breeding in the region [28]. Based on the documented benefits of organic acids in poultry nutrition, we hypothesized that supplementation with composite acidifying agents would enhance both production performance and gut health in 300-day-old BIAN chickens through improved nutrient utilization and beneficial microbiota modulation. This study therefore evaluated the effects of two concentrations of composite acidifiers (containing 2-hydroxy-4-methylthiobutyric acid, lactic acid, and phosphoric acid) on egg production, quality parameters, serum biochemistry, intestinal function, and cecal microbiota composition. By examining these integrated physiological responses, we sought to determine the optimal acidifier dosage and elucidate the mechanisms underlying performance improvements, providing evidence for implementing acidifier-based feeding strategies as sustainable alternatives to antibiotics in indigenous chicken production.

## 2. Materials and Methods

### 2.1. Test Materials and Test Design

The experimental protocols were conducted under the guidance of the Institutional Animal Care and Use Committee of Shanxi Agricultural University (No. SXAU2023045) for the humane care and use of animals in research. BIAN chicken (300-day-old) laying hens were provided by Shanxi Nongkang Xintuo Technology Development Co., Ltd. (Taiyuan, China). Liquid compound acidifier was used as a test additive, and its main components were 2-hydroxy-4-methylthiobutyric acid ≥ 30.0%, lactic acid ≥ 24.2%, and phosphoric acid ≥ 23.8%, which was manufactured by Novus International Co., Ltd. The product was used as provided and added via drinking water in the experimental groups.

The 900 BIAN chickens were housed in a three-tier battery cage system with galvanized wire cages measuring 60 cm (width) × 50 cm (depth) × 45 cm (height). Each cage housed 5 birds, providing 600 cm^2^ per bird. Each cage was equipped with nipple drinkers connected to independent waterlines for each replicate, allowing precise delivery of the acidifier treatments through drinking water. The house was maintained at 18–24 °C with a relative humidity of 55–65%, with 16 h of illumination daily at 15–20 lux intensity. Laying hens were fed a basal diet throughout the whole process, and the condition of the chickens was observed and recorded every day. The compound acidifier was added through drinking water, and each repetition was provided with an independent waterline. Each group was fed daily (fully automated feeding by machine), ad libitum food and water, and immunized according to normal immunization procedures. Nine hundred laying chickens were randomly selected and fed for 42 days. Prior to treatment allocation, birds were monitored for one week to confirm uniformity in laying rate, feed intake, and egg weight across all groups (*p* > 0.05). They were randomly divided into 3 treatments: control group: tap water (CON); test group A: CON + 0.05% compound acidifier; and test group B: CON + 0.20% compound acidifier. The laying rate of the 3 treatment groups was adjusted one week before the experiment, and 6 replicates were set up in each group; each replicate had 50 laying hens. The composition and nutritional components of the experimental basal diet are shown in Table 1.

### 2.2. Determination Index and Method

#### 2.2.1. Determination of Production Performance

During the 42-day experimental period, egg production was recorded daily at 18:00, egg weight was measured weekly, and feed intake was determined daily by recovering and weighing residual feed. The egg production, egg weight, and daily feed intake of each replication were counted. The egg production rate, daily feed intake, and feed-to-egg ratio were calculated. The egg laying rate (%) was calculated as follows: (total number of eggs laid/number of chickens × number of test days) × 100%. The average egg weight (g) was determined as follows: total egg weight/total egg number. The daily feed intake was calculated by subtracting the residual feed weight from the amount provided, then dividing by the number of hens: (feed provided-residual feed)/number of laying hens. The feed conversion ratio was calculated as follows: total feed intake/total egg weight.

#### 2.2.2. Determination of Egg Quality

At the end of the experiment, 15 eggs were randomly selected from each replicate of each treatment group, and 90 eggs were randomly selected from each group for egg quality determination. Egg quality index: egg weight, eggshell strength, eggshell thickness, egg shape index, and Haugh unit. Determination method: The egg shape index was measured with a vernier caliper, eggshell thickness was measured with an eggshell thickness tester (Robotmation, Tokyo, Japan), eggshell strength was measured with eggshell strength tester EFG0503 (Robotmation, Tokyo, Japan), and egg weight and Haugh unit were measured with egg quality analyzer EMT-7300 (Robotmation, Tokyo, Japan).

#### 2.2.3. Determination of Serum Biochemical and Antioxidant Indices

At the end of the experiment, 3 laying hens were randomly selected from each replicate for blood collection. In this experiment, the blood was collected from the vein below the wing and centrifuged, and the supernatant was stored at −20 °C for subsequent determination of indicators. The blood samples were used for serum biochemical analysis. Serum biochemical indicators: total protein (TP), albumin (ALB), glucose (GLU), triglyceride (TG), total cholesterol (TC), aspartate aminotransferase (AST), and other indicators. Determination method: Mindray BS-180 automatic biochemical analyzer was used. Serum antioxidant index: total antioxidant capacity (T-AOC). Colorimetry was used, and the test method was carried out according to the instructions for use of the kit produced by the Nanjing Institute of Bioengineering (Nanjing, China).

#### 2.2.4. Intestinal Digestive Enzyme Activity and Morphological Analysis

At the end of the experiment, the laying hens selected at the time of blood collection were slaughtered, and the chyme of the duodenum, jejunum, and ileum was collected in 4 mL sterile refrigerated centrifuge tubes and stored at -20 °C for later use. The test kit was purchased from Nanjing Jiancheng Institute of Biological Engineering (Nanjing, China). The following was performed:

(1) Determination of lipase activity: Accurately weigh chyme, add 2 steel balls according to the ratio of chyme mass to physiological saline of 1:4, then prepare homogenate under the condition of an ice water bath, homogenate at 2500 rpm (700× *g*, rotor radius 10 cm) for 10 min, then centrifuge at 3000 rpm (1000× *g*, rotor radius 10 cm) for 10 min, and carefully extract the supernatant to determine lipase activity. The test method was carried out according to the instructions for use of the kit.
(2) Trypsin and amylase activity determination: Accurately weigh chyme, prepare mixture with chyme mass and sample homogenate medium volume ratio of 1:9, add 2 steel balls, prepare homogenate under ice bath conditions, homogenize at 2500 rpm (700× *g*, rotor radius 10 cm) for 10 min, then centrifuge at 3000 rpm (1000× *g*, rotor radius 10 cm) for 10 min, and carefully extract supernatant to determine trypsin and amylase activity. The test method was carried out according to the instructions for use of the kit.

The duodenum, jejunum, and ileum of the test chickens were collected; the length was about 2 cm. Blood stains and contents were washed clean with 0.9% normal saline and fixed in fixing solution, which was prepared by 10% neutral buffered formalin solution–concentrated formaldehyde and phosphate buffer solution at a ratio of 1:9, and then paraffin sections were prepared. Hematoxylin and eosin (H&E) staining was used, followed by measuring the height and crypt depth of at least ten well-arranged villi and calculating the ratio of villi height to crypt depth. All histomorphometric data acquisition was conducted using Olympus Microscope (Olympus, Tokyo, Japan) and ImageJ software version 1.54g (National Institute of Health, Bethesda, MD, USA).

#### 2.2.5. Cecum Microbial Diversity Determination

At the end of the 42-day experimental period, 6 laying hens (342 days old) were randomly selected from each group. After slaughter, cecum contents were collected and put into sterile 5 mL cryopreservation tubes. They were quickly put into liquid nitrogen and then transferred to a −80 °C refrigerator for storage. Genomic DNA was extracted from cecal microorganisms using the Environmental Sample DNA Extraction Kit, and DNA quality was tested using 0.8% agarose gel electrophoresis. DNA samples are then sent for sequencing.

Sequencing method: After extracting the total DNA of the sample, design primers according to the conserved regions; add sequencing adapters to the ends of the primers; carry out PCR amplification; purify, quantify, and homogenize the products to form a sequencing library; carry out library quality inspection on the established library; and sequence the qualified library with Illumina Novaseq 6000. First, Trimmomatic v0.33 software was used to perform quality filtering on Raw Reads obtained by sequencing; then, cutadapt 1.9.1 software was used to identify and remove primer sequences to obtain Clean Reads without primer sequences; Usearch v10 software was used to splice the Clean Reads of each sample through overlap, and then length filtering was performed on the spliced data according to the length range of different regions. Using UCHIME v4.2 software, chimera sequences were identified and removed to obtain effective reads. Usearch software was used to cluster the reads at a similarity level of 97.0% to obtain OUT; QIIME V1.9.0 was used to evaluate the alpha diversity index of the samples. Using SILVA as the reference database, the naive Bayes classifier was used to annotate the feature sequences, and the taxonomic information corresponding to each feature was obtained. Then, the community composition of each sample can be counted at the phyla to genus level. The species abundance table at different taxonomic levels was generated by QIIME V1.9.0.

### 2.3. Data Statistics and Analysis

The data were processed with SPSS 25.0, using one-way ANOVA to compare means among the three treatment groups, followed by the Waller–Duncan post hoc test for multiple comparisons. The results are expressed as the “mean ± standard error”. The same or no superscript within the same line means no significant difference (*p* > 0.05); a different superscript means a significant difference (*p* < 0.05).

## 3. Results

### 3.1. Effect of Compound Acidifier on Production Performance of BIAN Chickens

As shown in Table 2, the compound acidifier had a significant effect on the average egg weight of BIAN chickens. The egg weight of test group A and test group B increased significantly (*p* < 0.05), compared with the control group, increasing by 1.82% and 4.6%, respectively. The laying rate of groups A and B increased slightly, but the difference was not significant (*p* > 0.05). The daily feed intake and feed/egg ratio of groups A and B showed no significant difference (*p* > 0.05).

### 3.2. Effect of Compound Acidifier on BIAN Chicken Egg Quality 

Table 3 shows that the compound acidifier had a significant effect on the shell strength and Haugh unit of the eggs. The Haugh unit values increased progressively with acidifier supplementation, with the control group showing 54.89 ± 2.10, the 0.05% acidifier group showing 64.11 ± 2.57, and the 0.20% acidifier group showing 73.26 ± 3.41, with all groups differing significantly from each other (*p* < 0.01). Eggshell strength was significantly higher in the 0.05% acidifier group (35.85 ± 1.16 N) compared to both the control (31.08 ± 1.22 N) and 0.20% acidifier groups (32.26 ± 0.80 N), which did not differ from each other (*p* < 0.01). No significant differences were observed for eggshell thickness or the egg shape index among treatments (*p* > 0.05).

### 3.3. Determination of Serum Biochemical Indices of BIAN Chickens Treated with Compound Acidifier

As can be seen from Table 4, the total antioxidant capacity (T-AOC) was significantly elevated only in the 0.20% acidifier group (11.97 ± 1.26 U/mL) compared to both the control (7.40 ± 1.11 U/mL) and 0.05% acidifier groups (7.57 ± 0.63 U/mL), representing a 61.76% increase (*p* = 0.010). Glucose levels differed significantly between the 0.05% acidifier group (11.27 ± 0.25 mmol/L) and the 0.20% acidifier group (5.98 ± 1.42 mmol/L), with the control group showing intermediate values (9.18 ± 1.68 mmol/L) that did not differ significantly from either treatment (*p* = 0.033). Total protein and globulin showed progressive dose-dependent increases across all three groups, with each group differing significantly from the others (*p* = 0.010). Specifically, total protein increased from 45.90 ± 2.01 mmol/L in the controls to 54.60 ± 2.85 mmol/L with 0.05% acidifier and 66.00 ± 3.20 mmol/L with 0.20% acidifier. Similarly, globulin increased from 29.44 ± 0.44 mmol/L in the controls to 37.78 ± 2.30 mmol/L and 48.72 ± 2.72 mmol/L in the 0.05% and 0.20% acidifier groups, respectively, representing increases of 28.33% and 65.49%. No significant differences were observed for albumin, triglyceride, total cholesterol, or aspartate aminotransferase among treatments (*p* > 0.05). From the overall effect, the additive amount of test group B is better than that of test group A. These changes occurred alongside stable values for other serum chemistry indices (including aspartate aminotransferase) and routine daily clinical observations during the trial, supporting a healthy physiological status across groups.

### 3.4. Effect of Compound Acidifier on Digestive Enzyme Activity of BIAN Chickens

As can be seen from Table 5, compared with the control, the lipase activity in duodenal contents (*p* < 0.05) and trypsin activity (*p* < 0.05) of test group A were significantly increased, while amylase activity showed no significant difference (*p* = 0.244), and amylase and lipase activities in jejunal contents were significantly increased (*p* < 0.05). Lipase activity in the duodenum and jejunum (*p* < 0.05) was significantly increased in group B. The trypsin activity of experimental group A was significantly higher than that of experimental group B (*p* < 0.05). Specifically, duodenal lipase activity increased from 0.81 U/gprot in the control group to 1.31 U/gprot in test group A, representing 61.73%. Similarly, duodenal trypsin activity increased by 24.74%, jejunal amylase activity increased by 37.43%, and jejunal lipase activity increased by 48.60% in test group A compared to the control. Lipase activity in the duodenum (98.77% increase) and jejunum (61.68% increase) was significantly increased in test group B (*p* < 0.05). The trypsin activity of experimental group A was significantly higher than that of experimental group B (*p* < 0.05).

### 3.5. Effect of Compound Acidifier on Intestinal Morphology of BIAN Chickens

As can be seen from Table 6, compared with the control group, test group B showed significant improvements in duodenal morphology. The duodenal crypt depth was significantly reduced from 203.71 μm (control) to 150.69 μm (test group B), a 26.02% reduction (*p* < 0.05). The villus-to-crypt ratio (V:C) significantly increased from 6.58 (control) to 9.51 (test group B), a 44.53% increase (*p* < 0.05). No significant changes were observed in jejunal or ileal morphology (*p* > 0.05). Test group A showed no significant effect on the intestinal morphology at any site compared with the control group (*p* > 0.05).

### 3.6. Effects of Compound Acidifier on Microbial Diversity in Cecum of BIAN Chickens

**Figure 1:**
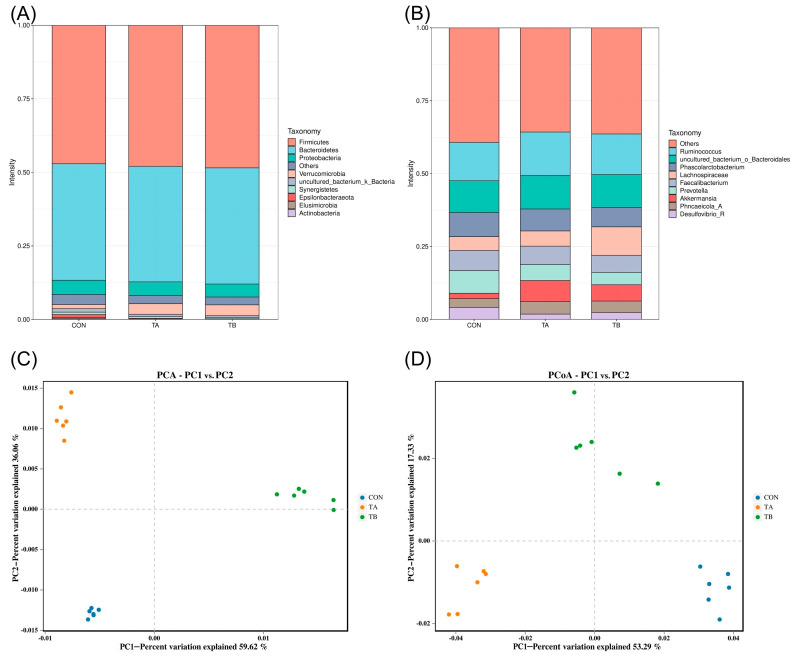
Effects of compound acidifier on cecal microbial community in BIAN chickens. (**A**) Relative abundance at phylum level. (**B**) Relative abundance at genus level. (**C**) PCA. (**D**) PCoA. CON: control; TA: CON + 0.05% compound acidifier; TB: CON + 0.20% compound acidifier.

Supplementation with the compound acidifier significantly modulated the cecal microbiota composition and diversity in laying hens. Alpha diversity analysis (Table 7) revealed that while the species richness remained consistent across groups (*p* = 0.173), the Shannon index was significantly lower in test group A (6.86 ± 0.02) compared to the control (7.17 ± 0.01) and test group B (7.12 ± 0.02) (*p* = 0.010), indicating altered bacterial community evenness. The Chao1 index showed statistically significant but minimal differences between both test groups (591.84 ± 2.27 and 591.84 ± 1.80) and the control (585.28 ± 1.16) (*p* = 0.043). The clear separation observed in the PCA and PCoA, along with substantial shifts in specific bacterial genera such as *Akkermansia* and Lachnospiraceae, indicates that acidifier supplementation induced meaningful changes in the microbial community composition despite the relatively stable alpha diversity metrics. Figure 1 illustrates the effect of the acidifier on the microbial community in the cecum of BIAN chickens. At the phylum level, the cecal microbiota was dominated by Firmicutes and Bacteroidetes, with additional presence of Proteobacteria and Fusobacteria. Taxonomic profiling at the genus level revealed substantial shifts in key bacterial populations, with *Akkermansia* showing marked increases from 1.8% in the control group to 7.2% in test group A and 5.6% in test group B. Similarly, Lachnospiraceae abundance increased from 4.7% in the control to 5.2% in test group A and notably to 9.7% in test group B. Principal component analysis (PCA) and principal coordinate analysis (PCoA) demonstrated clear separation between the treatment groups, with PC1 explaining 59.62% and 53.29% of the variation, respectively, indicating distinct microbiota profiles induced by acidifier supplementation. The bacterial community also harbored various other genera, including *Bacteroides*, *Faecalibacterium*, *Phascolarctobacterium*, and *Ruminococcus*, though their relative abundances showed less pronounced treatment effects. These findings indicate that compound acidifiers effectively modulate the cecal microbiome structure, particularly enhancing beneficial bacteria such as *Akkermansia*, which has been associated with improved gut health and metabolic functions in poultry.

## 4. Discussion

The present study demonstrated that dietary supplementation with a composite acidifier significantly increased the average egg weight in 300-day-old BIAN chickens, with test group B showing a remarkable 4.6% improvement compared to the control group. This finding is consistent with previous research showing that acidifiers enhance egg weight through improved nutrient digestibility and intestinal morphology [29,30]. The mechanism underlying this improvement involves acidifiers’ ability to lower the gastrointestinal pH, which enhances digestive enzyme activities and mineral absorption, particularly calcium and phosphorus utilization, crucial for egg formation [31,32]. Although the egg production rate showed no significant improvement in our study, the enhanced egg weight suggests that acidifiers primarily influence egg quality parameters rather than quantity in 300-day-old hens. At the laying stage, ovulation patterns are physiologically established, limiting the potential for increased egg numbers. However, the improved digestive enzyme activities and enhanced intestinal architecture enabled more efficient nutrient absorption, which birds preferentially allocated toward increasing individual egg mass rather than egg frequency. This nutrient partitioning pattern is consistent with findings in Japanese quails where acidifiers improved the egg weight and shell quality without significantly affecting the laying rate [33]. The lack of significant differences in the feed intake and feed-to-egg ratio between groups indicates that the improved egg weight resulted from enhanced nutrient utilization efficiency rather than increased consumption [34]. These results support the potential of composite acidifiers as effective alternatives to antibiotic growth promoters in laying hen production, offering a sustainable approach to improving egg quality while maintaining production efficiency [35]. The significant improvements in the Haugh unit and eggshell strength observed in test group A align with findings that composite acidifiers enhance calcium utilization and protein digestibility in laying poultry [36]. Enhanced Haugh unit values in both treatment groups corroborate recent evidence that organic acids improve albumen quality through increased nutrient absorption and antioxidant capacity [37]. The differential responses between groups suggest dose-dependent effects of acidifiers on mineral metabolism and shell gland function in BIAN chickens [38].

The present study demonstrated that dietary supplementation with composite acidifying agents significantly enhanced serum biochemical indicators in 300-day-old BIAN chickens, with test group B (65.49% globulin increase, 61.76% T-AOC elevation) showing superior effects compared to test group A (28.33% globulin increase). These findings align with recent studies reporting that organic acids influence serum biochemical parameters and the health status in poultry [39,40]. The marked elevation in serum globulin levels reflects enhanced immunoglobulin production, as globulins primarily consist of immunoglobulins (IgG, IgM, IgA) that constitute the humoral immune response. The mechanism by which composite acidifiers enhance globulin production likely involves multiple pathways. Acidifiers reduce the intestinal pH, creating conditions that favor beneficial bacteria like *Akkermansia* and Lachnospiraceae, which produce immunomodulatory metabolites including short-chain fatty acids (SCFAs). These SCFAs, particularly butyrate, activate G-protein-coupled receptors on intestinal epithelial cells and dendritic cells, triggering downstream signaling [41,42]. Additionally, the improved intestinal barrier function (evidenced by the increased villus-to-crypt ratio) reduces pathogenic bacterial translocation, decreasing inflammatory cytokine production while promoting regulatory T-cell responses that support optimal antibody production. The concurrent increase in total antioxidant capacity may further support immune function by reducing oxidative stress on immune cells, thereby preserving their capacity for antibody production [43,44]. The superior performance of test group B suggests that optimal acidifier dosage is crucial for maximizing immunomodulatory effects, as higher concentrations promote better intestinal pH regulation and nutrient absorption [45]. The enhanced antioxidant status observed in our study, as evidenced by the significant increase in total antioxidant capacity (T-AOC), demonstrates the beneficial effects of acidifiers on oxidative stress resistance. Previous research has suggested various pathways through which organic acids may enhance antioxidant capacity, including modulation of cellular signaling pathways and direct scavenging activities [46]. These improvements in immune and antioxidant parameters collectively contribute to a better health status and potentially enhanced production performance in BIAN chickens, supporting acidifiers as effective antibiotic alternatives in poultry production.

The composite acidifying agent significantly enhanced intestinal digestive enzyme activities in 300-day-old BIAN chickens, with test group A demonstrating a superior enzymatic response compared to test group B. The marked increases in lipase activity (61.73% and 48.60% in duodenal and jejunal contents, respectively) and trypsin activity (24.74% in duodenal content) in test group A align with studies showing that organic acids stimulate pancreatic secretion through pH-mediated secretin release [47]. The elevated amylase activity (37.43% in jejunal content) corroborates previous findings that acidifiers enhance carbohydrate digestion by promoting enzyme secretion [48]. Organic acids likely exert their effects by lowering the gastrointestinal pH, thereby activating pepsinogen to pepsin and triggering the release of cholecystokinin and gastrin, which subsequently stimulate pancreatic enzyme production [49]. The differential enzyme responses between test groups suggest dose-dependent effects on pancreatic function, with optimal acidifier concentrations maximizing proteolytic and lipolytic activities [50]. These enzymatic improvements facilitate nutrient digestion and absorption, potentially explaining the enhanced growth performance commonly observed with acidifier supplementation [51], thereby supporting acidifiers as effective alternatives to antibiotic growth promoters in BIAN chicken production. The composite acidifying agent demonstrated dose-dependent effects on intestinal morphology in 300-day-old BIAN chickens, with test group B showing significant improvements in duodenal architecture. The marked reduction in the crypt depth (26.02% decrease) and substantial increase in the villus-to-crypt ratio (44.53% elevation) in test group B align with studies demonstrating that organic acids enhance intestinal structure through pH modulation and antimicrobial activity [52]. The improved V:C ratio indicates enhanced absorptive efficiency and reduced epithelial cell turnover, consistent with findings that acidifiers promote intestinal maturation and nutrient absorption capacity [53]. The absence of morphological changes in test group A suggests that an optimal acidifier concentration is crucial for structural modifications, corroborating the dose-dependent effects reported in previous studies [54]. The decreased crypt depth likely reflects reduced pathogenic bacterial colonization and diminished inflammatory responses, as organic acids create an unfavorable environment for harmful microorganisms [55]. These morphological improvements are indicative of enhanced intestinal health and function, potentially explaining the superior performance outcomes commonly associated with acidifier supplementation [56].

The composite acidifying agent induced significant modulation of cecal microbiota composition through multiple interconnected mechanisms. The acidifiers directly lower the intestinal pH, which inhibits acid-sensitive pathogens through disruption of their cytoplasmic pH homeostasis as organic acids penetrate bacterial membranes in an undissociated form and dissociate intracellularly, causing metabolic dysfunction. This pH reduction selectively favors acid-tolerant beneficial bacteria like Lactobacillus and promotes *Akkermansia* growth, which thrives in mildly acidic mucin-rich environments. The increased *Akkermansia* abundance (from 1.8% to 7.2%) likely enhanced mucin turnover, producing acetate and propionate that serve as cross-feeding substrates for the enhanced Lachnospiraceae population (9.7% vs. 4.7% in control). The butyrate produced by Lachnospiraceae upregulates intestinal tight junction proteins and stimulates antimicrobial peptide secretion from Paneth cells, explaining the improved V:C ratio (44.53% increase), contributing to the observed 61.76% increase in antioxidant capacity [57]. This pattern contrasts with previous findings where higher-performing birds exhibited greater alpha diversity, suggesting that acidifiers specifically target certain bacterial populations rather than broadly increasing diversity [58]. The marked increase in *Akkermansia* abundance from 1.8% in the control to 7.2% in test group A and 5.6% in test group B was particularly noteworthy, as this genus has been associated with improved intestinal barrier function and metabolic health through mucin degradation and cross-feeding interactions with butyrate-producing bacteria [59]. The increased *Akkermansia* abundance observed in our study (from 1.8% in the control to 7.2% in test group A and 5.6% in test group B) may contribute to the improved egg production performance and intestinal health observed in BIAN chickens. *Akkermansia* species are known for their mucin-degrading capabilities and have been associated with enhanced intestinal barrier function and metabolic health through cross-feeding interactions with butyrate-producing bacteria [60]. The substantial enhancement in Lachnospiraceae abundance, particularly in test group B (9.7% vs. 4.7% in control), indicated improved fiber fermentation capacity and short-chain fatty acid production, as these bacteria are primary degraders of complex polysaccharides and major butyrate producers [61]. The synergistic interactions between Akkermansia and Lachnospiraceae create a beneficial metabolic network that enhances the observed improvements in egg weight and antioxidant capacity through improved mineral absorption and reduced intestinal inflammation. The clear separation between treatment groups was confirmed by both ordination methods: principal component analysis (PCA) showed that the first principal component (PC1) explained 59.62% of the variation, while principal coordinate analysis (PCoA) revealed that the first principal coordinate (PCo1) accounted for 53.29% of the variation. The consistency of group separation across these different analytical approaches strengthens the conclusion that acidifier supplementation created distinct microbial ecological niches [62]. These microbiota shifts aligned with the physiological improvements observed, as the enhanced populations of beneficial bacteria likely contributed to improved nutrient digestibility, immune function, and intestinal health through multiple mechanisms including competitive exclusion of pathogens, production of antimicrobial compounds, and modulation of host immune responses [63]. The maintenance of Firmicutes and Bacteroidetes dominance while selectively enhancing beneficial genera demonstrated that acidifiers preserved core microbiota stability while optimizing functional capacity [64]. The differential responses between test groups suggested that an optimal acidifier concentration was crucial for achieving the desired microbiota modulation, with test group B showing more pronounced beneficial changes in Lachnospiraceae abundance [65]. These microbiota alterations likely contributed to the observed improvements in digestive enzyme activities and intestinal morphology through enhanced production of microbial metabolites, particularly short-chain fatty acids, which serve as energy sources for intestinal epithelial cells and regulate gut homeostasis [66]. The comprehensive microbiome remodeling induced by composite acidifiers supports their role as effective alternatives to antibiotics in maintaining gut health and optimizing production performance in BIAN chickens.

The study shows that composite acidifiers significantly improve egg production performance and health in BIAN chickens, likely due to the metabolic effects of their primary components, such as organic acids, and their impact on gut health and immune function. HMTBa serves as a methionine source, supporting protein synthesis and related sulfur amino acid pathways in poultry. Lactic acid contributes to lumen acidification and antimicrobial control, which aligns with documented benefits of lactic acid systems in poultry. Phosphoric acid provides bioavailable phosphate in addition to its acidifying action, thereby aiding phosphorus availability for metabolism and eggshell formation. These metabolic processes underline the significant effects of compound acidifiers in enhancing poultry production and health, and further research into the detailed metabolic pathways involved will be valuable.

## 5. Conclusions

This study demonstrated that dietary supplementation with composite acidifying agents selectively improved specific production and physiological parameters in 300-day-old BIAN chickens. While the laying rate remained unaffected, the acidifier supplementation significantly increased the average egg weight (4.6% in test group B), enhanced egg quality parameters (Haugh unit and shell strength), and elevated serum antioxidant capacity (61.76% T-AOC increase). The treatment also markedly enhanced digestive enzyme activities (up to 61.73% lipase increase) and optimized intestinal morphology through a reduced crypt depth and an elevated villus-to-crypt ratio. These improvements suggest that composite acidifiers primarily influence egg quality and physiological health rather than overall production quantity in laying hens. The composite acidifiers induced beneficial modulation of the cecal microbiota composition, particularly increasing the abundance of health-promoting genera *Akkermansia* and Lachnospiraceae, which are associated with improved intestinal barrier function and short-chain fatty acid production. The dose-dependent responses observed between treatment groups indicate that the 0.20% acidifier concentration provided superior overall benefits across production, immunological, digestive, and microbiological parameters, though the 0.05% concentration showed specific advantages for eggshell strength and certain enzyme activities. Based on these comprehensive findings, the 0.20% concentration of composite acidifier (containing 2-hydroxy-4-methylthiobutyric acid, lactic acid, and phosphoric acid) delivered through drinking water is recommended for commercial BIAN chicken production as an effective alternative to antibiotic growth promoters, offering a sustainable approach to improving productivity and health status while maintaining intestinal homeostasis. Future research should evaluate acidifier efficacy over 6-12-month periods to establish long-term safety and economic viability in commercial production systems.

## Figures and Tables

**Table 1:** Basal diet composition and nutrient levels (air-dried basis).

Items	Contents (%)
Ingredients	
Corn	56.40
Soybean meal	25.50
Wheat bran	4.80
Soybean oil	2.34
Limestone	8.30
CaHPO_4_	1.50
L-Lys·HCl	0.04
L-methionine	0.14
L-threonine	0.04
L-tryptophan	0.01
NaCl	0.30
Choline chloride (50%)	0.10
Premix ^(1)^	0.53
Total	100.00
Nutrition level ^(2)^	
CP	16.58
Ca	3.76
AP	0.31
ME (MJ/kg)	11.12

Note: ^(1)^ The premix provides the following per kg of the diet: VA, 8000 IU; VD_3_, 1600 IU; VE, 5 IU; VK_3_, 4.00 mg; VB_1_, 0.80 mg; VB_2_, 2.50 mg; VB_6_, 1.50 mg; VB_12_, 0.004 mg; folic acid, 0.25 mg; nicotinic acid, 20.0 mg; pantothenic acid, 2.20 mg; biotin, 0.10 mg; Fe (FeSO_4_·H_2_O), 60.0 mg; Cu (CuSO_4_·5H_2_O), 8.0 mg; Mn (MnSO_4_·H_2_O), 60.0 mg; Zn (ZnSO_4_·H_2_O), 80.0 mg; Se (Na_2_SeO_3_), 0.30 mg; and I (KI), 0.35 mg. ^(2)^ ME is a calculated value, while the others are measured values.

**Table 2:** The effect of the composite acidifier on the production performance of BIAN chickens.

Item	Levels of Acidifier in Drinking Water			*p*-Value
	0.00%	0.05%	0.20%	
Laying rate	82.50 ± 0.86	83.96 ± 2.00	83.50 ± 1.96	0.83
Average egg weight	60.87 ± 0.78 ^b^	61.98 ± 0.80 ^ab^	63.67 ± 1.06 ^a^	0.029
ADFI	112.6 ± 0.8	113.5 ± 1.02	117.2 ± 1.01	0.92
Feed-to-egg ratio	2.06 ± 0.04	2.11 ± 0.1	2.12 ± 0.08	0.93

Note: Different lowercase letters following the data indicate significant differences (*p* < 0.05), and no letters or the same lowercase letters indicate no significance (*p* > 0.05).

**Table 3:** Effects of compound acidifier on egg quality characteristics of BIAN chickens.

Item	Levels of Acidifier in Drinking Water			*p*-Value
	0.00%	0.05%	0.20%	
Haugh unit (Ha)	54.89 ± 2.10 ^c^	64.11 ± 2.57 ^b^	73.26 ± 3.41 ^a^	0.01
Eggshell strength (N)	31.08 ± 1.22 ^b^	35.85 ± 1.16 ^a^	32.26 ± 0.80 ^b^	0.01
Eggshell thickness (mm)	0.32 ± 0.01	0.32 ± 0.01	0.33 ± 0.01	0.643
Egg shape index	1.32 ± 0.02	1.33 ± 0.02	1.33 ± 0.02	0.65

Note: Different lowercase letters following the data indicate significant differences (*p* < 0.05), and no letters or the same lowercase letters indicate no significance (*p* > 0.05).

**Table 4:** Results of determination of serum biochemical indices of BIAN chickens treated with compound.

Item	Levels of Acidifier in Drinking Water			*p*-Value
	0.00%	0.05%	0.20%	
T-AOC, U/mL	7.40 ± 1.11 ^b^	7.57 ± 0.63 ^b^	11.97 ± 1.26 ^a^	0.010
GLU, mmol/L	9.18 ± 1.68 ^ab^	11.27 ± 0.25 ^a^	5.98 ± 1.42 ^b^	0.033
TP, g/L	45.90 ± 2.01 ^c^	54.60 ± 2.85 ^b^	66.00 ± 3.20 ^a^	0.010
ALB, g/L	15.32 ± 0.99	16.82 ± 0.58	17.28 ± 0.71	0.206
GLB, g/L	29.44 ± 0.44 ^c^	37.78 ± 2.30 ^b^	48.72 ± 2.72 ^a^	0.010
TG, mmol/L	6.18 ± 1.46	10.23 ± 1.00	9.23 ± 1.63	0.133
TC, mmol/L	2.18 ± 0.53	2.81 ± 0.19	3.36 ± 0.51	0.795
AST, g/L	180.35 ± 11.18	170.07 ± 6.06	178.28 ± 16.91	0.822

Note: Different lowercase letters following the data indicate significant differences (*p* < 0.05), and no letters or the same lowercase letters indicate no significance (*p* > 0.05).

**Table 5:** The effect of the compound acidifier on the intestinal digestive enzyme activity of BIAN chickens.

Item	Levels of Acidifier in Drinking Water			*p*-Value
	0.00%	0.05%	0.20%	
Amylase (U/gprot)				
Duodenum	816.33 ± 105.33	975.2 ± 59.91	987 ± 53.8	0.244
Jejunum	977.33 ± 128.22 ^b^	1342.8 ± 153.36 ^a^	1151.5 ± 138.22 ^ab^	0.036
Ileum	993.5 ± 57.69	1124.2 ± 169.61	1093.67 ± 220.95	0.162
Lipase (U/gprot)				
Duodenum	0.81 ± 0.17 ^b^	1.31 ± 0.14 ^a^	1.61 ± 0.08 ^a^	0.010
Jejunum	1.07 ± 0.03 ^b^	1.59 ± 0.11 ^a^	1.73 ± 0.04 ^a^	0.010
Ileum	1.65 ± 0.14	1.71 ± 0.15	1.68 ± 0.07	0.112
Trypsin (U/mgprot)				
Duodenum	3121.12 ± 213.58 ^b^	3894.95 ± 351.54 ^a^	3318.35 ± 181.77 ^b^	0.010
Jejunum	4055.43 ± 880.08	4880.27 ± 666.92	4604.22 ± 629.30	0.200
Ileum	3106.59 ± 225.67	3202.19 ± 210.07	3295.74 ± 291.53	0.275

Note: Different lowercase letters following the data indicate significant differences (*p* < 0.05), and no letters or the same lowercase letters indicate no significance (*p* > 0.05).

**Table 6:** Effect of composite acidifier on intestinal morphometry of BIAN chickens.

Item	Levels of Acidifier in Drinking Water			*p*-Value
	0.00%	0.05%	0.20%	
Duodenum				
Villous length, μm	1341.80 ± 70.49	1210.20 ± 60.54	1365.75 ± 55.43	0.210
Crypt depth, μm	203.71 ± 19.13 ^a^	181.18 ± 21.19 ^ab^	150.69 ± 17.16 ^b^	0.025
V:C	6.58 ± 0.60 ^b^	7.17 ± 0.85 ^b^	9.51 ± 0.82 ^a^	0.030
Jejunum				
Villous length, μm	843.34 ± 31.49	863.37 ± 18.81	940.13 ± 50.19	0.166
Crypt depth, μm	125.09 ± 4.96	118.77 ± 5.14	131.20 ± 13.47	0.156
V:C	6.78 ± 0.30	7.34 ± 0.38	7.16 ± 0.57	0.346
Ileum				
Villous length, μm	701.20 ± 41.96	727.66 ± 16.31	715.09 ± 37.07	0.180
Crypt depth, μm	96.76 ± 6.47	97.52 ± 2.24	101.94 ± 7.94	0.125
V:C	7.24 ± 0.29	7.46 ± 0.26	7.02 ± 0.17	0.114

Note: Different lowercase letters following the data indicate significant differences (*p* < 0.05), and no letters or the same lowercase letters indicate no significance (*p* > 0.05).

**Table 7:** Effects of compound acidifier on alpha diversity index of cecal bacteria.

Diversity Index	Levels of Acidifier in Drinking Water			*p*-Value
	0.00%	0.05%	0.20%	
Species (s)	579.33 ± 0.95	583.00 ± 1.23	579.83 ± 1.8	0.173
Sequencing coverage (%)	99.97 ± 0.004	99.96 ± 0.004	99.96 ± 0.002	0.078
Shannon index	7.17 ± 0.01 ^a^	6.86 ± 0.02 ^b^	7.12 ± 0.02 ^a^	0.010
Simpson index	0.98 ± 0.01	0.97 ± 0.01	0.98 ± 0.01	0.051
Chao1 index	585.28 ± 1.16 ^b^	591.84 ± 2.27 ^a^	591.84 ± 1.80 ^a^	0.043
Ace estimate	583.91 ± 0.92	588.80 ± 1.32	586.67 ± 2.00	0.098
PD whole-tree lineage	35.26 ± 0.06	35.23 ± 0.05	35.29 ± 0.08	0.847

Note: Different lowercase letters following the data indicate significant differences (*p* < 0.05), and no letters or the same lowercase letters indicate no significance (*p* > 0.05).

## Data Availability

The data presented in this study are available on request from the corresponding author.
